# Medical Appointment in the Regular Army of the United States

**Published:** 1847-04

**Authors:** 


*Medical Appointment in the Regular Army of the United States.*—To persons desirous of entering the medical staff of the regular army, the following information from a responsible source will be acceptable:

It is prescribed by law that "no person shall receive the appointment of assistant surgeon in the army of the United States unless he shall have been examined and approved by an army medical board, to consist of not less than three surgeons or assistant surgeons, who shall be designated for that purpose by the Secretary of War."

Applications for permission to be examined for the appointment of assistant surgeon must be addressed to the Secretary of War; must state age and residence of the applicant; and must be accompanied by respectable testimonials of his possessing the moral and physical qualifications requisite for filling creditably the responsible station, and for performing ably the arduous and active duties of an officer of the medical staff. These proving satisfactory, invitations are accordingly sent to the applicants.

The medical board of examiners rigidly scrutinizes the pretensions of each candidate, taking into consideration his physical qualifications and moral habits, as well as his professional acquirements; and reports favourably upon no case admitting of a reasonable doubt, as the health and lives of the officers and soldiers are objects too important to be committed to ignorant and incompetent hands.

Section 8 of the army bill, approved February 11, 1847, authorizes the appointment of "two additional surgeons and twelve additional assistant surgeons in the regular army of the United States." After the promotion of two assistant surgeons to the advanced grade of surgeon, and the appointment of the candidates who were examined and found qualified by the last medical board, there will still remain nine appointments of assistant surgeons to be made under the provisions of the section just quoted.

An army medical board has accordingly been ordered to convene in the city of New York on the 15th of this month (March,) for the examination of such candidates as may be authorized by the Secretary of War to present themselves. Applicants from a distance are notified that, as the board will probably be in session for a month, they will be in time if they present themselves three weeks after the board shall assemble.—*Nat. Intel.*

